# Hexaminolevulinate blue light cystoscopy improves bladder cancer detection in comparison to white light cystoscopy: a prospective, comparative, within-patient controlled multicenter phase III bridging study in China

**DOI:** 10.3389/fruro.2025.1713128

**Published:** 2026-01-12

**Authors:** Hailong Hu, Jian Huang, Lulin Ma, Shudong Zhang, Jianming Guo, Xiuheng Liu, Yonglian Guo, Jin Wen, Hongxian Zhang, Shuai Jiang, Wang He, Cheng Liu, Xiaoliang Yuan, Monika Haefner, Bernd-Claus Weber, Kristine Young-Halvorsen, Hanzhong Li

**Affiliations:** 1 The Second Hospital of Tianjin Medical University, Department of Urology, Tianjin, China; 2 Sun Yat-Sen Memorial Hospital, Sun Yat-Sen University, Department of Urology, Guangzhou, China; 3 Peking University Third Hospital, Department of Urology, Beijing, China; 4 Zhongshan Hospital, Fudan University, Department of Urology, Shanghai, China; 5 Renmin Hospital of Wuhan University, Department of Urology, Wuhan, China; 6 The Central Hospital of Wuhan, Department of Urology, Wuhan, China; 7 Peking Union Medical College Hospital, Department of Urology, Beijing, China; 8 Jiangsu Yahong Meditech Co., Development Dept., Taizhou, China; 9 Richard Wolf GmbH, Clinical Affairs Dept., Knittlingen, Germany; 10 Richard Wolf GmbH, Development Image Proc. and Endoscopy Dept., Knittlingen, Germany; 11 Photocure ASA, Clinical Development and Medical Affairs, Oslo, Norway

**Keywords:** NMIBC, bladder cancer, CIS, blue light cystoscopy, hexaminolevulinate

## Abstract

**Background and objective:**

To compare hexaminolevulinate (HAL) blue light cystoscopy (BLC) with white light cystoscopy (WLC) in the detection of bladder cancer.

**Methods:**

Patients received intravesical HAL (Hexvix^®^) and underwent WLC before randomization to undergo high-definition BLC (System blue). Lesions identified in either WLC or BLC were evaluated by a blinded panel. The primary efficacy endpoint was the proportion of patients with histology-confirmed tumors (Ta, T1, or CIS) and with at least one such tumor found by BLC but not by WLC. The secondary endpoints included the detection of CIS, lesion detection rates, false-positive rate, and safety.

**Results:**

Of the 158 (160 screened patients) enrolled patients, 120 underwent WLC and were randomized (6 WLC, 114 BLC), and 97 were diagnosed with NMIBC. The mean age was 65.30 ± 12.18 years. Out of the 114 patients, 13 (11.4%) suffered from CIS; 84.6% (11/13) were detected with additional lesions by BLC; and 61.5% (8/13) were diagnosed solely by BLC. Compared with WLC, the proportion of patients with additional bladder cancer lesions detected by HAL BLC was 43.3% [(33.27%, 53.75%), *p* < 0.0001]. The proportion of patients with CIS lesions detected by HAL BLC and not by WLC was 9.6% (4.9%, 16.6%). The detection rates for CIS, Ta, T1, and T2–T4 tumors were 94.7%, 100%, 98.2%, and 100% for BLC and 42.1%, 76.1%, 91.2%, and 100% for WLC, respectively. The false-positive rates were 23.2% (19.2%, 27.7%) and 16.0% (11.9%, 20.8%) for BLC and WLC, respectively. A total of 95 patients (60.1%) reported 200 cases of AE, with 9 AEs being drug-related (fever, bladder pain, etc.). Nine device deficiencies (5.7%) occurred (eight quality issues and one device failure). No AEs and SAEs led to discontinuation.

**Conclusions:**

In the setting of modern high-definition equipment, HAL BLC significantly improves the detection of bladder cancer with favorable safety.

## 1 Introduction

Bladder cancer is the ninth most common cancer worldwide (1). In 2022, 614,298 new cancer cases and more than 220,000 deaths were attributed to bladder cancer worldwide, while in China, the number of new cases and deaths reached 92,883 and 41,367, respectively (1). Approximately 75% of bladder cancer patients present with non-muscle-invasive bladder cancer (NMIBC) at the initial diagnosis (2). NMIBC is characterized by high recurrence rates, ranging from 50% to 80% recurrence risk within 5 years of diagnosis (3, 4).

White light cystoscopy (WLC) is the standard method used to visualize the bladder mucosa. WLC has been shown to have limitations in the detection of lesions, especially of flat, non-papillary lesions, such as carcinoma *in situ* (CIS) and small or multifocal lesions (5–7). Therefore, enhanced cystoscopy is recommended to improve detection and reduce recurrence of NMIBC (5, 8–10). Photodynamic diagnosis is an enhanced cystoscopy technique that uses a photosensitizer to improve visualization of bladder lesions. Hexaminolevulinate (HAL) causes the accumulation of photosensitive protoporphyrin IX (PPIX) in bladder lesions. When illuminated during blue light cystoscopy (BLC), these porphyrins emit red light, improving the detection of bladder cancer lesions in general and CIS in particular (11–17). Correspondingly, HAL BLC has been shown to improve the quality of TURBT in primary and recurrent patients by detection of clinically relevant lesions, enabling more complete resection corresponding to a change in management (6, 18, 19). As a result, BLC has a favorable impact on recurrence rates and outcomes (20–22).

Although the efficacy of HAL BLC is well-established, the advantages were demonstrated in comparison to traditional WLC using older visualization equipment and standard definition monitors. Recent technological advancements in endoscopic imaging, including high-definition (HD) imaging, improved visualization with filters, and image processing, have significantly enhanced the visualization even with WLC. Hence, the marginal value of using BLC over state-of-the-art WLC needs to be re-evaluated (23).

There are no recent studies that have re-evaluated whether BLC can still maintain its advantage over WLC when utilizing the most current equipment. The aim of this study was to compare HAL BLC with WLC in the detection of bladder cancer using the most current HD cystoscopic equipment. The study also served to evaluate this technology in Chinese patients to enable governmental approval for the routine use of BLC in China.

## 2 Materials and methods

### 2.1 Study design, setting, and period

This prospective, comparative, within-patient controlled, multicenter, phase III clinical trial was conducted at seven tertiary hospitals in China between November 2022 and July 2023 (NCT05600322).

**Figure 1:**
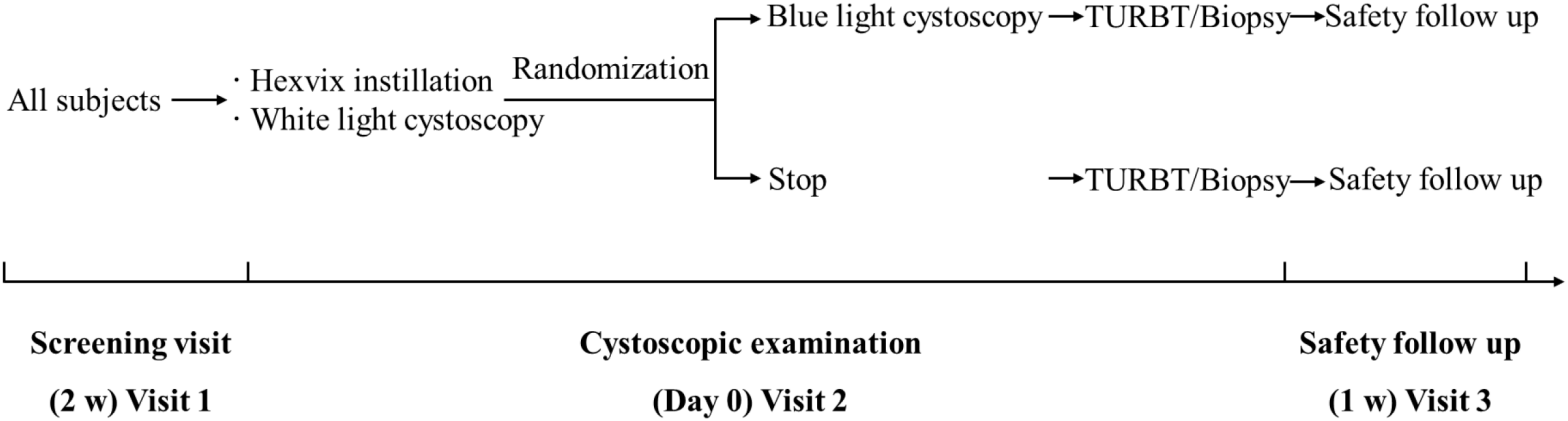
Study design.

The study included three visits (**Figure 1**). After screening, all patients received intravesical HAL (50 mL, 8 mmol/L, Hexvix^®^, Photocure ASA, Oslo, Norway) for 1 h. All patients then underwent WLC with inspection of the bladder and mapping of all lesions and suspicious areas in the operating room under anesthesia. After the completion of the WLC examination, a call is placed to the central coordinating center to obtain the randomization group. Patients randomized to undergo BLC were then subjected to BLC, whereas those not assigned to BLC received standard treatment and were included only in the safety population.

The risk of being randomized to not undergo the BLC procedure was used to mitigate potential performance bias. In this setup, 5% of the subjects were randomly selected not to receive blue light cystoscopy. The aim was to ensure that the urologists remained thorough in their white light inspections, knowing that it was not certain for a specific patient that enhanced detection with blue light would be available. The investigators were blinded to the randomization scheme, i.e., they did not know about the probability of being randomized to not undergo BLC. The randomization also happened after the WLC bladder inspection was completed. If randomized to continue, the patients underwent BLC with inspection of the bladder and mapping of the lesions (System blue, Richard Wolf GmbH). The approach used to minimize bias was based on previously published studies by Daneshmand et al. (16) and Jocham et al. (18).

All suspicious lesions observed were recorded, including the number, size, and location, and were subsequently resected or biopsied. For patients randomized to BLC, the completeness of the resection was checked under blue light. Any additional lesions noted on BLC only and missed on WLC were also biopsied separately and evaluated. Pathological evaluation of biopsies and resected specimens was conducted by an independent central panel of pathologists who were blinded to the cystoscopic evaluation and whether the specimen was observed under WLC, BLC, or both. Patients were assessed for safety after 1 week, which constituted the final study visit. Subsequent treatment and management were carried out according to routine medical practice (**Figure 1**).

### 2.2 Study population and eligibility criteria

Adult patients with suspected or confirmed bladder cancer were enrolled in the study after giving their written informed consent. Patients suspected of having bladder cancer were those who had symptoms and signs of bladder cancer, such as hematuria and imaging evidence, and were judged by clinicians to require cystoscopy and TURBT in the operating room. Confirmed bladder cancer patients were those who had been diagnosed with bladder cancer based on pathological evidence and required further cystoscopic treatment. Patients with gross hematuria or those who received intravesical therapy of any kind in the past 6 weeks or with porphyria or hypersensitivity to HAL or pregnant or breastfeeding were excluded.

### 2.3 Sample size and sampling procedure

The primary efficacy endpoint of this study was the proportion of patients with histology-confirmed tumors (Ta, T1, or CIS) who had at least one such tumor found by HAL BLC but not by WLC. A proportionate increase of ≥10% of patients detected as having at least one more lesion on BLC compared to WLC was considered clinically significant, and if the actual proportion was 20%, a total of 94 subjects with pathologically confirmed Ta, T1, and CIS stage bladder cancer were required at a one-sided significance level of 0.025 with 80% power based on an exact test for one proportion.

### 2.4 Data collection and laboratory procedure

At the start of the study, a minimum of four patients per center were enrolled as training subjects who underwent WLC and HAL BLC with lesion resection and safety follow-up. The purpose was to train study personnel in the protocol-defined blue light cystoscopic procedure and blue light equipment, which is not currently available in China. These training patients were excluded from the efficacy analyses. This training phase was designed similarly to the approach described by Daneshmand et al. (16).

The primary endpoint was the proportion of subjects with ≥1 histologically confirmed tumor (Ta, T1, or CIS) detected by HAL BLC but not by WLC. The secondary endpoints included 1) the proportion of patients with ≥1 CIS lesions detected by HAL BLC and not by WLC; 2) the lesion detection rate of PUNLMP, CIS, Ta, T1, and T2–4 by HAL BLC and WLC; and 3) the proportion of patients with false-positive lesions detected by HAL BLC and WLC. Safety was evaluated separately for both the drug product and the medical device. The drug safety assessment included reporting of adverse events (AEs), including serious AEs (SAEs), treatment-emergent AEs, and related AEs. Device safety endpoints included AEs, SAEs, related AEs, and device deficiencies. Safety was assessed in all patients undergoing the respective interventions, including the training patients.

### 2.5 Data analysis

The primary efficacy endpoint of this study was the proportion of patients with histology-confirmed tumors (Ta, T1, or CIS) who had at least one such tumor found by HAL BLC but not by WLC. The secondary endpoints were presented with descriptive statistics with the 95% CI. All statistical analyses were performed by SAS 9.4. A *p*-value <0.025 (unilateral) was considered statistically significant.

### 2.6 Ethical considerations

The multicenter study was coordinated by Peking Union Medical College Hospital (PUMCH), which obtained ethical approval from its Ethics Committee (No. HS2022068). The study was conducted in accordance with the ICH GCP-E6, local regulatory requirements, and the Declaration of Helsinki. Each participating center also obtained local ethics approval before study initiation.

## 3 Results

**Figure 2:**
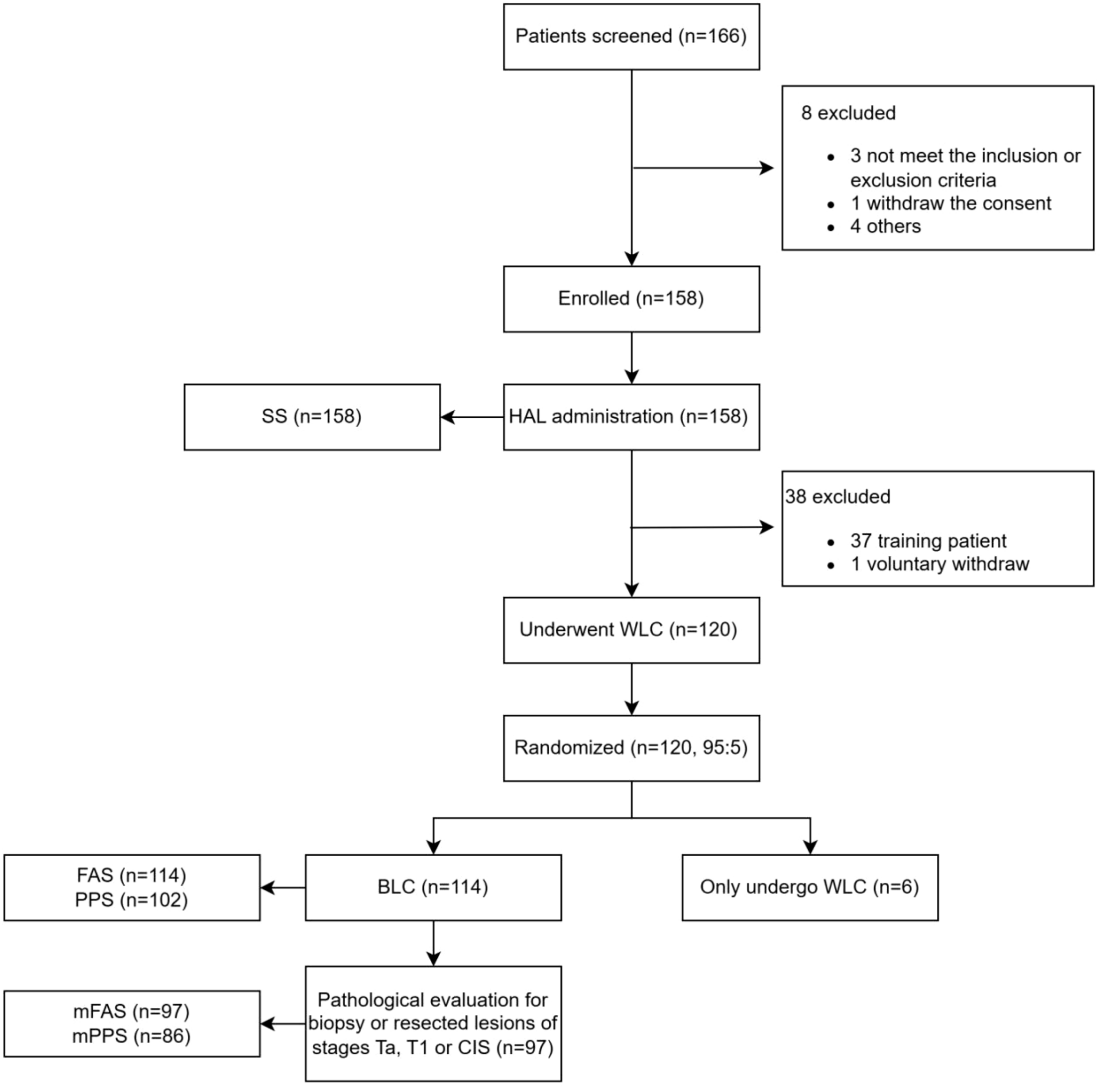
CONSORT diagram. HAL, hexaminolevulinate; WLC, white light cystoscopy; BLC, blue light cystoscopy; FAS, the full analysis set, including all patients who received HAL BLC who were not training patients; mFAS, the modified FAS, was a subset of FAS and included those patients from FAS who had histologically confirmed diagnosis of Ta, T1, or CIS; PPS, the per protocol set population included those in the FAS population without major protocol deviations. Such major deviations included 1) failure to fulfill inclusion criteria/exclusion criteria but still entered into the study, 2) failure to retain the HAL solution for at least 30 min, and 3) other protocol violations that were thought to affect the result of the cystoscopy or the histopathological assessment; mPPS, the modified PPS population included those patients in PPS who had histologically confirmed diagnosis of Ta, T1, or CIS; SS, the safety set included all subjects who received HAL (including training patients).

Of 166 screened patients, 158 patients were enrolled and received HAL in this study. Thirty-seven patients were training patients, 120 patients underwent WLC and were randomized, six patients were randomized to WLC, 114 patients underwent BLC, and 97 patients were diagnosed with NMIBC (**Figure 2**). The mean age of the patients was 65.30 ± 12.18 years. Of the 114 subjects, 80.7% were men and 36.8% had a history of bladder cancer, among whom 21.4% (9/42) had a history of CIS and 73.8% (31/42) had a history of high-grade Ta or T1 disease (**Table 1**). There were 33 (28.9%) subjects with a history of TURBTs.

**Table 1:** Patient characteristics.

Parameter	HAL BLC patients (*n* = 114)	HAL BLC patients with confirmed NMIBC (*n* = 97)
Age (mean ± SD, years)	65.30 ± 12.18	66.00 ± 11.89
Males [*n* (%)]	92 (80.7)	78 (80.4)
Height (mean ± SD, cm)	168.30 ± 7.37	168.10 ± 7.21
Weight (mean ± SD, kg)	68.65 ± 12.41	68.11 ± 12.19
BMI (mean ± SD, kg/m^2^)	24.13 ± 3.28	23.98 ± 3.27
Any history of bladder cancer [*n* (%)]	42 (36.8%)	32 (33.0%)
Any history of CIS (*n*)	9	6
Any history of high-grade Ta or T1 disease (*n*)	31	22

There were 97 patients with NMIBC, with 42 patients [43.3% (33.27%, 53.75%), *p* < 0.0001] having at least one confirmed lesion found by BLC but not WLC.

Of the 114 patients, 11.4% (13/114) of patients had CIS, and 9.6% [11/114 (4.9%, 16.6%)] had one or more additional CIS lesions detected by BLC. Among the 13 patients with CIS, 84.6% (11/13) showed at least one additional confirmed CIS lesion found by BLC but not by WLC. Of the patients with CIS, 61.5% (8/13) were diagnosed only by HAL BLC.

Among the 114 patients, a total of 306 lesions of PUNLMP, CIS, Ta, T1, and T2–T4 were detected using either HAL BLC or WLC (**Table 2**). HAL BLC detected 18 out of 19 CIS lesions (94.7%), while WLC detected only 8 (42.1%). All 226 Ta lesions were detected by HAL BLC, while WLC detected 172 (76.1%). Among 57 T1 lesions, 56 were detected by HAL BLC (98.2%) and 52 by WLC (91.2%). All 4 T2–T4 lesions were detected by both methods. The false-positive detection rates by HAL BLC and WLC were 23.2% (95% CI: 19.2%, 27.7%) and 16.0% (95% CI: 11.9%, 20.8%), respectively (**Table 3**). For all efficacy endpoints, the supporting analyses from the per-protocol analysis showed similar results (**Tables 2**, **3**).

**Table 2:** The detection rates of different bladder cancer types (PUNLMP, CIS, Ta, T1, T2–4).

Lesion type	Total number of lesions (*n*)	HAL BLC, *n* [% (95% CI)]	WLC, *n* [% (95% CI)]
HAL BLC patients (*n* = 114)			
PUNLMP	0	–	–
CIS	19	18 [94.7% (74.0%, 99.9%)]	8 [42.1% (20.3%, 66.5%)]
Ta	226	226 [100.0% (98.4%, 100.0%)]	172 [76.1% (70.0%, 81.5%)]
T1	57	56 [98.2% (90.6%, 100.0%)]	52 [91.2% (80.7%, 97.1%)]
T2–T4	4	4 [100.0% (39.8%, 100.0%)]	4 [100.0% (39.8%, 100.0%)]
Per protocol (*n* = 102)			
PUNLMP	0	–	–
CIS	13	12 [92.3% (64.0%, 99.8%)]	4 [30.8% (9.1%, 61.4%)]
Ta	194	194 [100% (98.1%, 100%)]	151 [77.8% (71.3%, 83.5%)]
T1	45	44 [97.8% (88.2%, 99.9%)]	42 [93.3% (81.7%, 98.6%)]
T2–T4	3	3 [100% (29.2%, 100%)]	3 [100% (29.2%, 100%)]

CIS, carcinoma *in situ*; HAL BLC, hexaminolevulinate blue light cystoscopy; PUNLMP, papillary urothelial neoplasm with low malignant potential; WLC, white light cystoscopy.

**Table 3:** The false-positive detection rates of HAL BLC and WLC.

Characteristics	HAL BLC	WLC
HAL BLC patients (*n* = 114)		
Number of lesions (*n*)	396	281
Number of false-positive lesions (*n*)	92	45
Proportion, % (95% CI)	23.2 (19.2, 27.7)	16.0 (11.9, 20.8)
Per protocol (*n* = 102)		
Number of lesions (*n*)	332	236
Number of false-positive lesions (*n*)	79	36
Proportion, % (95% CI)	23.8 (19.3, 28.7)	15.3 (10.9, 20.5)

HAL BLC, hexaminolevulinate blue light cystoscopy; WLC, white light cystoscopy.

Ninety-five (60.1%) of the 158 patients who were administered HAL and underwent WLC or BLC reported a total of 200 AEs of which 193 emerged after the interventions. There were eight patients with nine AEs potentially related to the drug, including pyrexia (*n* = 4), bladder pain (*n* = 1), hematuria (*n* = 1), urethral syndrome (*n* = 1), constipation (*n* = 1), and urinary tract infection (*n* = 1). No subjects experienced device-related AEs. For both drug and device, no SAEs were reported and no AEs led to discontinuation. During the clinical trial, 9 out of 158 subjects experienced nine cases of device deficiencies, resulting in an incidence rate of 5.7%. The deficiencies included eight cases of quality issues (seven cases with the same device and one case with a different device) and one case of device failure. None of the device deficiencies or quality issues resulted in any medical procedures, diseases, or injuries to the subjects, nor caused any AEs or SAEs. In addition, they had no impact on image quality or the primary study outcomes.

## 4 Discussion

This study is the first within-patient controlled clinical trial to show that HAL BLC is superior to WLC in the detection of NMIBC using modern LED 4K HD equipment in a Chinese population. Compared with WLC, HAL BLC could detect additional NMIBC in 43% of patients. HAL BLC detected additional CIS lesions in 84.6% (11/13) of patients with CIS compared with WLC.

Recently, a Cochrane review (24) demonstrated a 40% reduction in the relative risk of recurrence and a 31% reduction in the relative risk of progression of HAL BLC compared with WLC. A recent cohort study confirmed this impact and associated the benefit with more intensive and definitive therapy, suggesting that BLC helps identify patients for the most appropriate therapy, resulting in improved outcomes (25).

The proportion of patients with confirmed cancer was high in our study (80.8%, 97/120). This is likely a reflection of the study setting and the proportion of patients in our cohort who were enrolled based on a suspicion of primary cancer. The study was conducted in large tertiary (class 3A) hospitals in China, where TURBT was performed under anesthesia in the operating room. Patients were generally admitted only after outpatient diagnostic confirmation, leading to a cohort largely composed of pre-confirmed bladder cancer cases enriched through cytology- and imaging-based preselection at tertiary centers.

It is known that HAL BLC can improve the detection of NMIBC in various settings and populations (12–14, 26–32). Our study confirmed these findings in a Chinese population, which has not been previously reported. The overall detection benefit of HAL BLC was comparable to earlier experiences (43.3% vs. 16%–49%) (12, 29–31), particularly in patients undergoing TURBT in the operating room. Similarly, Hermann et al. reported that 49% of patients had additional residual lesions detected under blue light TURBT, consistent with our findings. Although the use of modern LED 4K HD systems was expected to improve WLC performance, the LED blue light mode still provided additional diagnostic benefit, especially in our tumor-enriched population undergoing TURBT in the operating room. Further validation will be needed in larger and more diverse patient populations in the future.

Notably, the detection rate of CIS with HAL BLC was 94.7%, compared with 42.1% with WLC. These results are equivalent to or surpass those reported in other trials. The rate of additional detection of CIS was higher than seen earlier in our study (12). Given the limited sample size (*n* = 13), this finding should be interpreted with caution, but it does emphasize the particular value of CIS detection using BLC. The improved detection is associated with a reduction in long-term outcomes. The detection of CIS is critically important due to the high risk of progression of this disease if left untreated. The detection benefit in our trial should be viewed in the context of the underlying population. Our trial enrolled a higher proportion of primary patients compared to earlier clinical trials, accompanied by a smaller proportion of CIS patients and a predominantly Chinese population. In line with our results, a recent real-world evidence study suggests that the efficacy of HAL BLC is improved in an Asian population (33). It is known that there is a higher prevalence and incidence of bladder cancer in Asia compared to other regions and that the outcome of bladder cancer is worse in Asia. It is suggested that this is due to a higher-risk population at the time of diagnosis. In line with the efficacy of HAL BLC, the benefit has been seen to be larger in higher-risk populations.

One of the potential drawbacks of HAL BLC is the increased false-positive rate. In our study, the false-positive rate was analyzed at the lesion level, rather than at the patient level. We found that the false-positive rate of HAL BLC was 23.2%, compared with 16.0% of WLC, which were not statistically different in line with the false-positive rates of HAL-BLC and WLC from other studies (6, 12, 34), despite the high prevalence of cancer in our cohort. Smaller differences in false-positive rates between studies are thought to reflect differences in surgeon training and experience, as well as, most importantly, the patient population.

We did not observe any serious adverse events related to the use of HAL or the BLC equipment. The results align with previous studies that reported a low incidence of adverse events with HAL BLC, which is expected after transurethral surgery (35). Our study supports that HAL BLC is a safe and well-tolerated procedure. We believe that improved detection and earlier diagnosis of high-risk lesions, such as CIS, may contribute to improved management and treatment decisions in such patients, leading to better outcomes.

## 5 Strengths and limitations

A novel aspect of our study was the use of modern LED 4K HD equipment for both WLC and HAL BLC. Previous controlled studies have used conventional standard definition equipment for WLC, and it has been proposed that LED 4K HD equipment could enhance WLC performance and reduce the difference between WLC and HAL BLC. However, our results showed that HAL BLC still had a significant advantage over WLC in the detection of NMIBC and CIS. Moreover, the LED 4K HD equipment may also improve HAL BLC performance by providing clearer and brighter images of fluorescent lesions, especially in the presence of blood or inflammation. In addition, the advantages of System blue compared to other modern equipment probably contributed to the improved detection rates. Therefore, we believe that the combination of HAL BLC and LED 4K HD equipment represents the optimal modality for bladder cancer detection.

This study only focused on the additional tumor detection rate and did not examine long-term benefits such as recurrence endpoints. The study population is relatively heterogeneous, lacking distinction or specific requirements for those with prior tumor history, initial onset, recurrence, or a history of CIS. Given that this is a bridging trial and considering the study’s objectives, these limitations are acceptable. These factors should be considered when interpreting the generalizability of our findings.

## 6 Conclusion

In the setting of modern LED 4K HD equipment, HAL BLC significantly improved the detection of NMIBC and especially CIS compared with WLC in Chinese patients with suspected or diagnosed bladder cancer, with a favorable safety profile. The results confirm and support the use of HAL BLC, also when novel, HD cystoscopic equipment is available, in line with guideline recommendations.

## Data Availability

The original contributions presented in the study are included in the article/supplementary material. Further inquiries can be directed to the corresponding authors.
